# Supplementary material for: Polarization Insensitive, Broadband, Near Diffraction-Limited Metalens in Ultraviolet Region
Source: Nanomaterials (Basel). 2020 Jul 23;10(8):1439. doi: 10.3390/nano10081439 (PMC7466348; doi:10.3390/nano10081439)
Supplement: Supplementary file 1 [file nanomaterials-10-01439-s001.pdf]

Supplementary material: Polarization Insensitive, Broadband, Near Diffraction-Limited Metalens in Ultraviolet Region

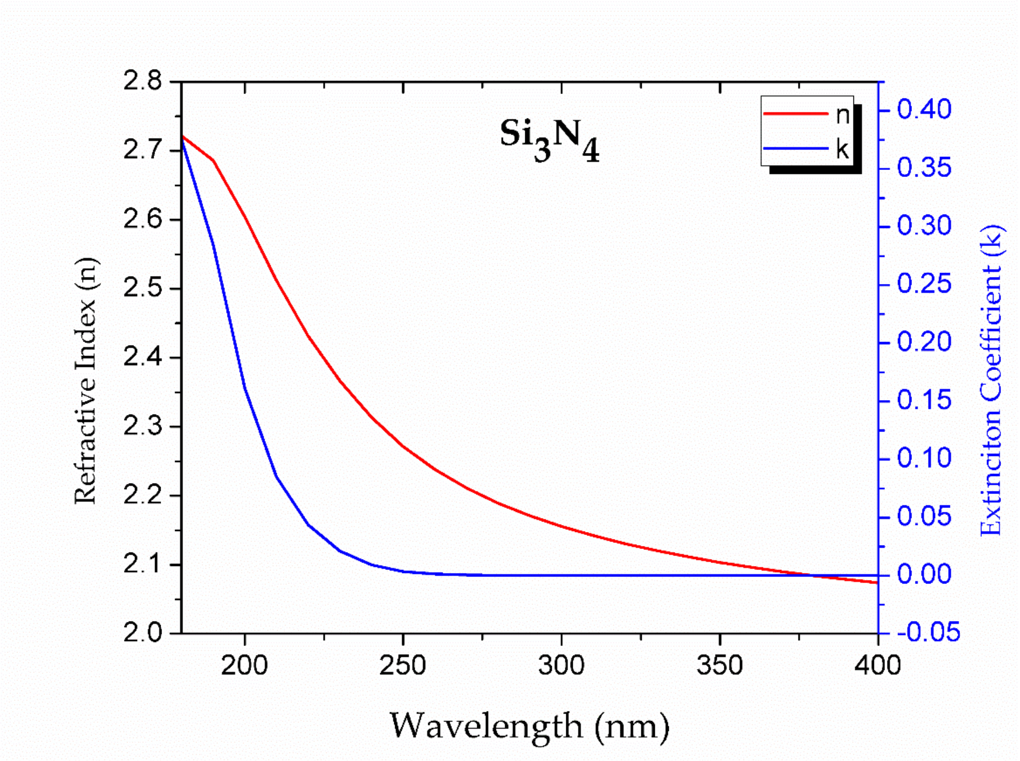

Figure S1. The real and imaginary part of the refractive index of Si_3N_4 utilized in this study, displaying a high refractive index ($n > 2$) and exceptionally low loss ($k = 0$) over a broad UV range.
